# Climate Clever Clovers: New Paradigm to Reduce the Environmental Footprint of Ruminants by Breeding Low Methanogenic Forages Utilizing Haplotype Variation

**DOI:** 10.3389/fpls.2017.01463

**Published:** 2017-09-05

**Authors:** Parwinder Kaur, Rudi Appels, Philipp E. Bayer, Gabriel Keeble-Gagnere, Jiankang Wang, Hideki Hirakawa, Kenta Shirasawa, Philip Vercoe, Katia Stefanova, Zoey Durmic, Phillip Nichols, Clinton Revell, Sachiko N. Isobe, David Edwards, William Erskine

**Affiliations:** ^1^Centre for Plant Genetics and Breeding, The University of Western Australia, Crawley WA, Australia; ^2^School of Agriculture and Environment, The University of Western Australia, Crawley WA, Australia; ^3^Institute of Agriculture, The University of Western Australia, Crawley WA, Australia; ^4^Centre for Personalised Medicine for Children, Telethon Kids Institute, Subiaco WA, Australia; ^5^Murdoch University, Perth WA, Australia; ^6^School of Biological Sciences, The University of Western Australia, Crawley WA, Australia; ^7^Institute of Crop Science, The National Key Facility for Crop Gene Resources and Genetic Improvement, Chinese Academy of Agricultural Sciences Beijing, China; ^8^Kazusa DNA Research Institute Kisarazu, Japan; ^9^Department of Agriculture and Food Western Australia, South Perth WA, Australia

**Keywords:** greenhouse gas emissions, ruminant enteric methanogenesis, genetic and genomic analyses, forage crops, natural variation, selecting haplotypes

## Abstract

Mitigating methane production by ruminants is a significant challenge to global livestock production. This research offers a new paradigm to reduce methane emissions from ruminants by breeding climate-clever clovers. We demonstrate wide genetic diversity for the trait methanogenic potential in Australia’s key pasture legume, subterranean clover (*Trifolium subterraneum* L.). In a bi-parental population the broadsense heritability in methanogenic potential was moderate (*H*^2^ = 0.4) and allelic variation in a region of Chr 8 accounted for 7.8% of phenotypic variation. In a genome-wide association study we identified four loci controlling methanogenic potential assessed by an *in vitro* fermentation system. Significantly, the discovery of a single nucleotide polymorphism (SNP) on Chr 5 in a defined haplotype block with an upstream putative candidate gene from a plant peroxidase-like superfamily (TSub_g18548) and a downstream lectin receptor protein kinase (TSub_g18549) provides valuable candidates for an assay for this complex trait. In this way haplotype variation can be tracked to breed pastures with reduced methanogenic potential. Of the quantitative trait loci candidates, the DNA-damage-repair/toleration DRT100-like protein (TSub_g26967), linked to avoid the severity of DNA damage induced by secondary metabolites, is considered central to enteric methane production, as are disease resistance (TSub_g26971, TSub_g26972, and TSub_g18549) and ribonuclease proteins (TSub_g26974, TSub_g26975). These proteins are good pointers to elucidate the genetic basis of *in vitro* microbial fermentability and enteric methanogenic potential in subterranean clover. The genes identified allow the design of a suite of markers for marker-assisted selection to reduce rumen methane emission in selected pasture legumes. We demonstrate the feasibility of a plant breeding approach without compromising animal productivity to mitigate enteric methane emissions, which is one of the most significant challenges to global livestock production.

## Introduction

Greenhouse gas (GHG) emissions are of major concern globally. Among heat-trapping gasses, methane is one of the most potent, about 25 times more than CO_2_, and contributing nearly as much to global warming as all other non-CO_2_ GHGs combined (EPA, 2010). As with other GHGs, the concentration of methane has risen and is now more than twice the level of the early 1800s (Kelly, 2014). Approximately 60% of current methane emissions are anthropogenic and include emissions from landfills, agriculture, and coal mining (EPA, 2010).

Methane is an unavoidable by product of the fermentation of feed in many animals, particularly ruminants (Naqvi and Sejian, 2011). The world’s population of domesticated ruminants produces about 15% of total methane emissions (Moss et al., 2000) and this also represents a substantial loss (2–15%) of gross energy intake (Johnson et al., 1993), by reducing the conversion of feed energy to metabolizable energy. Thus, the inhibition of ruminant methanogenesis provides the dual benefits of mitigating GHG emissions and using the energy consumed for production more efficiently (Patra and Saxena, 2010).

Methane production is influenced more by feed characteristics than animal genetic factors (EPA, 1995). The chemical composition of feed, in particular, is a major driver of ‘methanogenic potential’ (the amount of methane produced by rumen microbes during fermentation of feed), and dietary supplementation with fat and plant secondary metabolites have been shown to reduce enteric methanogenesis (Grainger and Beauchemin, 2011; Tang et al., 2014). As the response to direct selection of animals for low methanogenic potential in the rumen is likely to be slow (Shi et al., 2014), the methanogenic potential trait in pastures is a strong candidate for marker-assisted selection (MAS).

Variation between pasture species for *in vitro* microbial fermentability and methanogenic potential in the rumen ranged dramatically with the lowest methane-producing species, *Biserrula pelecinus* L., producing 90% less methane (4 mL CH_4_ g^-1^ dry matter incubated) than the highest methane-producing species, *Trifolium spumosum* L. (51 mL CH_4_ g^-1^ dry matter incubated) (Durmic et al., 2010; Banik et al., 2013a). Furthermore, intraspecific variation for rumen methanogenic potential has been reported within biserrula (*Biserrula pelecinus* L.) (Banik et al., 2013b) and subterranean clover (*Trifolium subterraneum* L.) (Banik et al., 2012) and there is potential to breed these legumes for more efficient fermentation. Among annual clovers, subterranean clover makes the greatest contribution globally to livestock feed production and soil improvement and 54 cultivars have been released in Australia, where it has been sown over 29 million ha (Nichols et al., 2013). Subterranean clover consists of three subspecies (ssp. *subterraneum*, ssp. *brachycalycinum*, and ssp. *yanninicum*) (Katznelson and Morley, 1965), with each adapted to different soil types. It is also used as a reference species for genetic and genomic studies within the genus *Trifolium*, as it is diploid (2n = 16), predominantly inbreeding, and has a well-assembled and annotated genome (Hirakawa et al., 2016; Kaur et al., 2017). Elucidation of the genetic basis in subterranean clover for *in vitro* microbial fermentability and enteric methanogenic potential is needed to understand these traits in the more genetically complex, but globally significant, perennial pasture legumes, white clover (*T. repens* L.) and red clover (*T. pratense* L.). It also complements genomic studies in the model annual legume *Medicago truncatula* L. and the important perennial fodder legume, lucerne (*M. sativa* L.) (Ghamkhar et al., 2011).

Since subterranean clover genotypes differ in their methanogenic potential *in vitro* (Banik et al., 2012), there is potential to breed for more efficient fermentation. The study was first to estimate the broadsense heritability of methanogenic potential in subterranean clover. Then because it was considered likely that the response to direct selection for methanogenic potential in the rumen would be slow, due to environmental variation attributable to the animal host and its rumen microbial population, the study aimed to identify genomic regions associated with methanogenic potential and to explore the feasibility of selecting haplotypes of alleles for reduced enteric methane production. We considered that the identification of genetic markers, for use in MAS, would provide an efficient and cost-effective means for breeders to select pasture cultivars with low methanogenic potential.

## Materials and Methods

### Plant Materials and Experimental Design

#### (i) F_2_-Derived Lines from a Bi-parental Cross

The F_3_ seeds were obtained from 176 individual F_2_ subterranean clover plants from the cross 92S80 (cv. Woogenellup × cv. Daliak). These parents were initially chosen as they differ widely in a range of agronomic and morphological characters (Ghamkhar et al., 2011). There was significant (*P* < 0.05) variability in the methanogenic potential amongst the two parents, ranging between 37.08 and 34.14 mL of CH_4_ per gram of DM supplied respectively (Supplementary Table S1). The experimental trial (sown in 2009) was conducted in two phases using a *p/q* partial replication design (Cullis et al., 2006). In Phase I, the parents and F_2_ progeny lines were grown in the glasshouse, while Phase II was conducted in the laboratory after leaf harvest for *in vitro* fermentability tests (IVFTs).

In Phase I, the experimental units were pots, 204 in total, comprising: (i) the two parents, Daliak and Woogenellup, replicated three times; (ii) 20 random F_3_ lines, replicated twice; and (iii) the remaining 158 F_3_ lines, which were replicated once. In Phase II, a separate randomization was used for the IVFT test which extended over 3 days. To assess inter-day variability, 24 of the 158 single-pot plant samples of F_3_ lines were sub-divided for analysis on different days bringing the total of plant samples to 228. Each of these 228 biological samples was subdivided into triplicates for IVFT analysis for assay replication.

#### (ii) A Diversity Panel of Core Collection Lines and Cultivars

The second experimental trial (sown in 2011) was grown in the field at Shenton Park, Western Australia (31°57′ S, 115°50′ E) and assayed in the laboratory. A panel of 124 *T. subterraneum* genotypes (Supplementary Table S2) was selected for the study, which included 97 core collection accessions (Nichols et al., 2013) and 27 diverse Australian cultivars. The core collection was developed by K. Ghamkhar, R. Appels, and R. Snowball to represent the genetic diversity within the world collection of >10,000 phenotypes (Nichols et al., 2013; Ghamkhar et al., 2014). Selection of the core collection followed the methodology of Ghamkhar et al. (2008) to identify a subset of 760 lines, on the basis of (i) diversity for eco-geographical data from their sites of collection; and (ii) agro-morphological data obtained by the Australian Trifolium Genetic Resource Centre (ATGRC) of the Department of Agriculture and Food Western Australia (DAFWA). DNA was then extracted from leaf material of each short-listed line and 48 single sequence repeat (SSR) primers, spread across each of the eight subterranean clover chromosomes, were selected from the results of Ghamkhar et al. (2011) to identify the most diverse lines. Analysis using MSTRAT software (Gouesnard et al., 2001) to optimize maximum diversity within the minimum number of lines, resulted in an optimum core collection of 97 lines, covering 80.1% of the genetic diversity within the whole subterranean clover collection.

Seeds of the 124 diversity panel lines were obtained from the ATGRC. All accessions were replicated twice in the field for biological replication and again in triplicate in the laboratory for assay replication. A row-column design with two-directional blocking was generated for the field and laboratory.

All designs were generated using the experimental design software DiGGer (Coombes et al., 2002) in R software (Johnson et al., 1993).

### Propagation and Harvest of Plant Material

Ten plants per line were grown in both experiments. Seeds for the 2009 experiment were sown into pots (22 cm diameter) in a phytotron maintained at 20/15°C day/night temperatures with 60% humidity. Group C *Rhizobium* inoculum, in the form of clustered clay granules (ALOSCA Technologies Pty Ltd.), was applied 7 days after sowing.

Seeds for the 2011 experiment were sown into hydrated Jiffy-9 peat pots (Jiffy Products Ltd., Norway) in a glasshouse. Three seeds were sown per pot, which were randomly thinned to a single seedling per pot 20 days later. Group C *Rhizobium* inoculum (Nodulaid, BASF Australia Ltd) was sprinkled onto peat pots 7 days after sowing and watered in gently. Peat pots were watered daily, while soluble fertilizer (Phostrogen, Bayer CropScience Ltd) was watered on weekly at a rate of 5 g/10 L. Seedlings were transplanted to the field into a moist, weed-free seedbed, described as a loam-dressed sand (Nichols et al., 2009), with pH (CaCl_2_) 6.5. The trial site was hand-weeded and irrigated by overhead sprinklers as required. Other site details and management are the same as described in Nichols et al. (2009).

The edible plant parts (leaves and stems less than 10 cm long) were harvested before flowering for *in vitro* fermentability testing. The material was then freeze-dried in a Bench-top Freeze Dryer (VirTis, Germany) and ground (Glen Creston, Stanmore, England) to pass through a 1.0 mm screen. Material was stored at room temperature in sealed containers for further analysis.

### Phenotyping for Methanogenic Potential Using an *In Vitro* Fermentability Test (IVFT) and Statistical Analyses

The methanogenic potential was examined in an *in vitro* batch fermentation system commonly used to examine plants and their extracts with rumen fluid (Cardozo et al., 2005; Busquet et al., 2006; Bodas et al., 2008). We followed the protocol by Durmic et al. (2010). Briefly, 1 day before the experiment, 0.1 g of plant material or oaten chaff was weighed in bellco tubes and transferred to an anaerobic chamber (Coy anaerobic chamber; 80% N_2_:10% CO_2_:10% H_2_) to expel oxygen from the tubes. On the experimental day, rumen fluid was collected from two fistulated sheep fed a general maintenance diet consisting of lupins and oaten chaff (1 kg oaten chaff + 250 g lupins + 25 g mineral mix) 2 h after feeding. This study was approved by and the donor sheep were cared for in accordance with the guidelines The University of Western Australia (UWA) Animal Ethics Committee (AUP number RA/3/100/1171). An electric vacuum pump fitted with a plastic tube was used to collect rumen fluid and then transported to the laboratory where it was strained through cheesecloth to remove large particles. After straining, the rumen fluid was pooled, transferred into the anaerobic chamber, and buffered to pH 7.2 using McDougall’s buffer (McDougall, 1948); 10 mL of this mix was dispensed into the prepared bellco tubes. Assay controls included a negative control (buffered rumen fluid only, NC) and a positive control (buffered rumen fluid + 0.1 g oaten chaff, PC). Inside the chamber, the tubes were sealed with rubber stoppers, crimped and then incubated for 24 h at 39°C, with constant shaking at 50 rpm, to mimic the rumen environment. At the end of the incubation period, tubes were placed in a water bath at 39°C, and the contents were transferred to an exetainer tube (Labco, United Kingdom) for methane analysis by gas chromatography. Methane concentration in the gas sample was determined using a concentration by gas chromatography (Bruker 450 GC, Bruker Technologies, Australia) with two packed columns and a Compass CDS data acquisition software (Bruker Technologies, Australia). The methanogenic potential of the plant was expressed as total methane produced per gram dry matter of substrate incubated (mL/g DM incubated) (Soliva et al., 2008).

A linear mixed model was used for the analysis of the two-phase experiments (Smith et al., 2006). Typically, in a multi-phase experiment, possible trends associated with residuals for each of the phases are modeled. In this study, spatial trends in the glasshouse/field and temporal trends in the laboratory were considered. The model accounted for block structure (biological replicates), spatial variation in the field, laboratory day variation, the possible correlation of ordered laboratory measurements within a day, the linear trend of ordered measurements within a day if present, and methane control values (fitted as a covariate).

The first phase component in the model accounted for possible spatial variation in the glasshouse/field, in particular, the possible presence of linear *row* or *column* effects, local trends, and some extraneous variation if present (Gilmour et al., 1997; Stefanova et al., 2009). The second phase component included the blocking structure in the laboratory, possible linear trends, and a covariance structure linked to the *order* of the samples within the *day*. Additionally, two covariates were included, positive and negative controls for *methane* and *gas*, respectively, measured each day.

Random effects in each model were predicted using empirical best linear unbiased prediction (E-BLUP), and fixed effects were estimated using empirical best linear unbiased estimation (E-BLUE). The obtained prediction error variance matrix for the variety effects was used to calculate broad-sense heritability (Falconer, 1960) for each trial and each response.

All statistical analyses were performed using ASREML-R (Butler et al., 2009) and R software^1^. The adjusted (predicted) mean values for methanogenic potential were used for quantitative trait loci (QTL) and genome-wide association study (GWAS) analyses.

### High-Density SNP Linkage Map and QTL Analysis

The high-density single nucleotide polymorphism (SNP) linkage map was constructed using MultiPoint 3.3^2^ as described in Hirakawa et al. (2016). QTL mapping was conducted using the high-density SNP linkage map anchored to the high-quality chromosome-level genome assembly (Kaur et al., 2017) by the integrated genetic analysis software QTL IciMapping v4.0^3^ (Meng et al., 2015). QTL with both additive and dominant genetic effects were screened using an inclusive composite interval mapping (ICIM) approach implemented in QTL IciMapping v4.0 (Zhang et al., 2008). Missing phenotypes were deleted using the ‘Deletion’ command in the software. The walking speed was set at 1 cM. A suitable probability for entering marker variables in stepwise regression was chosen so that the variation explained by the model approximated the trait heritability. The regression model was then used for background genetic variation control in the ICIM QTL mapping. The LOD was calculated using 1,000 permutations with the Type 1 error being 0.05, and significant QTLs were defined accordingly.

### Genotyping the Diversity Panel and Genome-Wide Associations

The population structure for GWAS was of two sub-populations: the first sub-population comprised 27 cultivars released in Southern Australia for grazing; while the second sub-population of 97 accessions was a core germplasm collection – a stratified sample of the world collection of *T. subterraneum*. The core collection was formed by a two-step selection process using ecological data and stratified proportional strategy followed by combined datasets and a maximizing strategy to best represent the entire collection (Ghamkhar et al., 2008).

Genomic DNA (gDNA) was extracted from a single plant from each of the 97 accessions of the subterranean clover core collection and the 27 diverse Australian cultivars, and sequenced. High-quality whole-genome resequencing (WGRS) data were generated for all 124 accessions and cultivars using paired-end sequencing libraries with an insert size of approximately 550 bp sequenced on an Illumina HiSeq 2000 sequencer. A total of 13.89 billion paired-end resequencing reads were generated with 5.27 billion reads uniquely mapped to the advanced genome assembly (Tsub_Refv2.0) (Kaur et al., 2017) using BWA (Li and Durbin, 2009). SNPs were identified using samtools and bcftools (Li et al., 2009; Li, 2011). The resulting SNPs were filtered by removing SNPs with at least one heterozygous allele, those with an MAF ≤ 5%, and those that were not present in at least one individual. Consecutive SNPs were merged using PLINK v1.9 (Purcell et al., 2007; Chang et al., 2015) into haplotype blocks if their *r*^2^-values were above 0.8. Linkage Disequilibium was visualized using Haploview v4.2 (Barrett et al., 2005).

The GWAS was conducted via a mixed linear model in TASSEL v5.2.30 (Bradbury et al., 2007) using four PCs and a kinship matrix as covariates as reported by TASSEL. Q-values were calculated using the R-package *q*-value (Storey et al., 2015). The Manhattan plot was drawn with qqman (Turner, unpublished).

### Marker-Trait Association Studies and Putative Candidate Gene Analysis

Phenotypic information obtained from the cross 92S80 (cv. Woogenellup × cv. Daliak) and the diversity panel of 124 core collection lines and cultivars was associated with specific regions in the advanced assembly (Tsub_Refv2.0) (Kaur et al., 2017) using QTL and GWAS analyses, respectively, as described above to map significant associations. Each significant marker-trait association (MTA) resulting from the GWAS was checked for any overlaps with haplotype blocks with *r*^2^-values above 0.8. In which case, 25-bp upstream and downstream from the SNP were extracted from the reference and used as input for primer3 v2.3.7 (Koressaar and Remm, 2007; Untergasser et al., 2012) (settings: primer product size 250–500, primer optimum size 300, primer minimum temperature 55°C, optimal temperature 57°C, maximum temperature 60°C).

Putative candidate genes were proposed for each significant MTA by extracting the genes upstream, downstream or overlapping with GWAS candidate SNPs, or genes located between the left and right markers of candidate QTLs.

## Results

### Variation and Heritability of Methanogenic Potential

The methanogenic potential measured in the IVFT was assessed using a linear mixed model fitting procedure (Butler et al., 2009). High correlation values for methane production were found among laboratory triplicates within the 2009 (*r*-values: 0.686, 0.672, and 0.759) and 2011 (*r*-values: 0.845, 0.793, and 0.860) measurements. Furthermore, the broad-sense heritability (Falconer, 1960) calculated for the 2009 trial (*H*^2^ = 0.4) reflected the complexity of the assayed trait.

### Marker-Trait Associations

The phenotyping information for methanogenic potential (Supplementary Tables S1, S2) was associated with specific regions in the high-density SNP linkage map. A significant association [*P* < 0.0002 at a threshold logarithm of odds (LODs) of threshold 2.5 defined by permutation tests] for methanogenic potential, *Q_MP_LG8*, was identified on Linkage Group 8 (LG8) with the phenotypic data available (**Table 1**). *Q_MP_LG8* was identified as a significant QTL accounting for 7.8% of the variation in methanogenic potential. Within the bi-parental cross, the ‘Daliak’ alleles had a positive additive effect (increased methanogenic potential) (**Table 1**).

**Table 1:** Significant genomic associations identified using the phenotyping data for the quantitative trait locus (QTL) mapping in the bi-parental cross 92S80 segregating for methanogenic potential (mL/g DM).

QTL position	Chr	Left marker	Right marker	Size of the region (bp)	PVE (%)	LOD	Add	Dom	Candidate genes	Candidate gene ID	Length	Functional annotation
73	8	Tsud_sc00224.00_ 395828	Tsud_sc00303.00_ 209352	231,687	7.8	2.6089	-0.8236	1.9512	11	TSub_g26967	1134	DNA-damage-repair/ toleration DRT100-like protein
										TSub_g26968	909	Uncharacterized protein
										TSub_g26969	1320	Suppressor-of-white-APricot splicing regulator
										TSub_g26970	1062	Transcription factor bHLH122-like protein
										TSub_g26971	507	Disease resistance protein
										TSub_g26972	372	Disease resistance protein
										TSub_g26973	1929	Flowering time control FY-like protein
										TSub_g26974	558	Ribonuclease H
										TSub_g26975	474	Ribonuclease T2 family protein
										TSub_g26976	1839	Far1-related sequence 10-like protein
										TSub_g26977	1008	Uncharacterized protein


**FIGURE 1:**
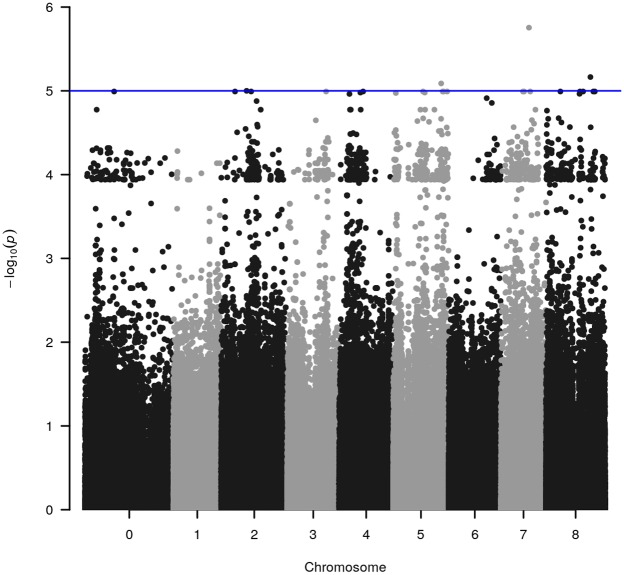
Significant marker-trait associations (MTAs) for methanogenic potential (mL/g DM) detected through genome-wide association analysis using the diverse germplasm panel of *Trifolium subterraneum.* The *y*-axis in each graph represents –log_10_*P* for the *P*-value of the MTA, while chromosomes are indicated on the *x*-axis. The blue line marks the threshold for genome-wide significance (*P*-value = –log_10_*P* > 5.0) considered as significantly associated.

WGRS data from the diversity panel were analyzed with phenotyping data collected from the GWAS method using TASSEL v5.2.30 for estimating MTAs (**Supplementary Figure S1**). The GWAS resulted in four markers, which reached suggestive *P*-values below 1e^-5^ on chromosomes 2, 5, 7, and 8 (**Figure 1** and **Table 2**). The SNP located on Chr 5 was located in a haplotype block containing six other SNPs with a total length of 27.63 Kbp.

**Table 2:** Significant genomic associations identified using the phenotyping data for the diverse germplasm panel in genome-wide association study (GWAS).

GWAS SNP position	Chr	UNADJ *P*-value	Q-values	Marker r^2^	^∗∗^Alleles	Overlapping gene	Upstream gene [distance from SNP]	Downstream gene [distance from SNP]	Candidate genes	Candidate gene ID	Length	Functional annotation
26105839	2	9.9926E-06	0.065898056	0.20808	G/T,A		TSub_g5136 [3505 bp]	TSub_g5137 [2619 bp]	2	TSub_g5136	1410	Uncharacterized protein
										TSub_g5137	690	Uncharacterized protein
^∗^49188043	5	0.000008162	0.065898056	0.21212	G/A,C		TSub_g18548 [22314 bp]	TSub_g18549 [1150 bp]	2	TSub_g18548	366	Plant peroxidase-like superfamily
										TSub_g18549	717	Lectin receptor protein kinases (LecRKs)
28784592	7	1.7606E-06	0.065898056	0.24317	G/A,T	TSub_g24126			1	TSub_g24126	2007	Esterase/lipase/ thioesterase family protein
45480260	8	6.8428E-06	0.065898056	0.21565	C/G,A		TSub_g27846 [12050 bp]	TSub_g27847 [3060 bp]	2	TSub_g27846	828	Tubulin beta-1 chain
										TSub_g27847	1005	Transmembrane protein


*^∗^Haplotype block; ^∗∗^alleles: G/T,A means that G is reference, T,A are alternative alleles in the population.*

### Associating SNPs to Gene Models and PCR-ready Markers to Track Haplotype Variation

Putative candidate genes were proposed for each significant MTA by extracting the genes upstream, downstream or overlapping with GWAS candidate SNPs, or genes located between the left and right markers of candidate QTLs.

The *Q_MP_LG8* at 231.7 Kbp long contained 11 of the genes suggested as putative candidates for this trait. Of these, there was a DNA-damage-repair/toleration DRT100-like protein (TSub_g26967), a suppressor-of-white-APricot splicing regulator (TSub_g26969), a transcription factor bHLH122-like protein, two disease resistance proteins (TSub_g26971, TSub_g26972), two ribonuclease proteins (TSub_g26974, TSub_g26975), a flowering time control FY-like protein (TSub_g26973) and a far1-related sequence 10-like protein (TSub_g26976) in addition to two uncharacterized proteins (TSub_g26968 and TSub_g26977) (**Table 1**).

**FIGURE 2:**
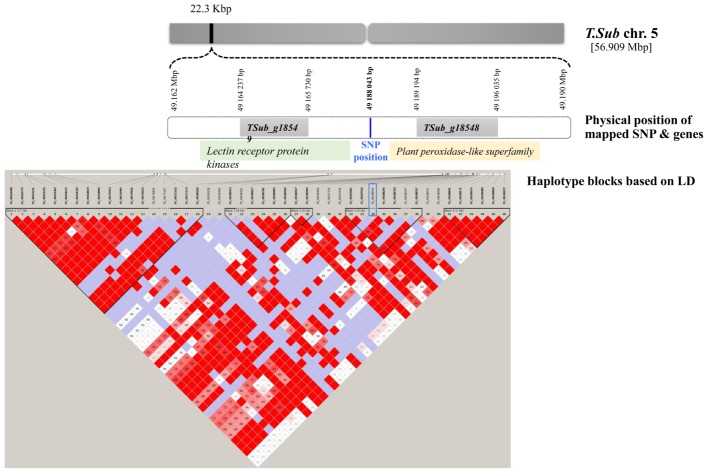
Scheme of the genome region with the haplotype block on Chr 5 in *T. subterraneum* (advanced Tsub_Rv2.0) showing the physical position of the methanogenic potential (mL/g DM) candidate genes and markers.

For the GWAS SNPs, one suggestive SNP was located at the first base of an esterase/lipase/thioesterase protein family gene (TSub_g24126 on Chr 7). On Chr 8, we identified an upstream tubulin beta-1 chain (TSub_g27846) and a downstream transmembrane protein (TSub_g27847) at a distance of 12,050/3,060 bp respectively, from the suggestive SNP. The SNP located on Chr 5 was mapped in a haplotype block with TSub_g18548 (plant peroxidase-like superfamily) upstream at 22,314 bp and TSub_g18549 (lectin receptor protein kinase) downstream at 1,150 bp (**Figure 2** and **Table 2**). Being in the haplotype block, these two genes are the most stable and promising candidates for designing molecular markers to track haplotype variation for this complex trait. The haplotype block containing the MTA SNP on Chr 5 with a total length of 27,629 bp was used to design PCR-ready markers for MAS for this complex trait (Supplementary Table S3).

### Linking Variation in Methanogenic Potential to Subspecies, Origin and Climate Data

The 124 diverse germplasm panel entries for methanogenic potential in the field ranged from 22.4 to 33.7 mL CH_4_/g DM. The two lowest values were ssp. *subterraneum* (22.4 mL CH_4_/g DM) and ssp. *brachycalycinum* (23.3 mL CH_4_/g DM), both collected from Italy (Supplementary Table S2). The diversity panel was analyzed using one-way ANOVA to test for differences in methanogenic potential between the means of the germplasm collection and the Australian cultivars, the means of the three subspecies, and accessions from different countries of origin but no differences were identified (**Supplementary Figures S2A–C** and Table S4). Similar analyses were run to identify any significant associations of methanogenic potential with the available passport data from collection sites (latitude, longitude, altitude, soil pH) and 19 BioClim climatic variables, as described in Ghamkhar et al. (2014), but no significant associations were found (**Supplementary Figure S3** and Table S5).

## Discussion

Given the imperative to reduce the methanogenic potential of ruminant feed and the likely sizeable environmental effect on its phenotypic expression, this study was designed to assess the heritability of methanogenic potential and to study the feasibility of a marker-assisted approach to its selection. The intermediate/low broadsense heritability of *H*^2^ = 0.4 in the bi-parental population emphasized the need to explore the marker approach. Variation in methanogenic potential in a segregating bi-parental population was localized to a QTL region in LG8 and explained 7.8% of the phenotypic variation. This genomic region at a sequence level implicated 11 putative candidate genes, including the DNA-damage-repair/toleration DRT100-like protein (TSub_g26967), linked to modulating the severity of DNA damage induced by the production of secondary metabolites considered important in enteric methane production. A plant having more of this or a variant of this that minimizes DNA damage by secondary compounds may be able to ‘handle’ more of the secondary compounds which ultimately then influences the rumen microbial organisms. Also implicated are three disease resistance genes (TSub_g26971, TSub_g26972, and TSub_g18549), a suppressor-of-white-APricot splicing regulator (TSub_g26969), a transcription factor bHLH122-like protein and ribonuclease proteins (TSub_g26974, TSub_g26975). These genes are good indicators for elucidating the genetics in subterranean clover of *in vitro* microbial fermentability and enteric methanogenic potential.

Use of a broad diversity panel for GWAS has a major advantage over the bi-parental approach, as it spans the entire genetic diversity of the forage, whereas the bi-parental approach focuses on a strictly limited sample of genetic material. From GWAS the most significant discovery was the SNP located on Chr 5, which was mapped in a haplotype block with a TSub_g18548 (plant peroxidase-like superfamily) upstream gene and a TSub_g18549 (lectin receptor protein kinase) downstream gene. The plant peroxidase-like superfamily performs a variety of biosynthetic and degradative functions. Among the sub-families, Class I includes intracellular peroxidases present in fungi, plants, archaea and bacteria, called catalase-peroxidases, that can exhibit both catalase and broad-spectrum peroxidase activities, depending on the steady-state concentration of hydrogen peroxide. Class II includes ligninase and other extracellular fungal peroxidases, while Class III comprises classic extracellular plant peroxidases, such as horseradish peroxidase (Veitch, 2004). Class III plant peroxidases are actively involved in the biosynthesis of terpenoid indole alkaloids in *C. roseus* (Sottomayor et al., 2004). The significant MTA with this TSub_g18548 plant peroxidase-like gene family in the present study has established the first link at the genomic level of defined metabolic pathways regulating plant secondary metabolites and fat-enriched diets implicated in methane metabolism.

The lectin receptor protein kinases contain legume lectin motifs originally found in the seeds of leguminous plants (Sharon and Lis, 1990). Legume lectins are well-characterized in terms of their three-dimensional structure, sugar–ligand interaction, and specificity. Legume lectins can bind various disaccharides such as glucose-mannose, galactose-*N*-acetylgalactosamine, fructose, chitobiose, and other complex sugars (Loris et al., 1998). It is well-documented in the literature that methane production in the animal gut may decline if starch or digestible nutrients escape rumen fermentation (Ellis et al., 2010, 2012; Dijkstra et al., 2011) and feeding high-sugar grasses alters the pattern of rumen fermentation (Ellis et al., 2012). Thus, increasing the soluble carbohydrate content in animal feed could rectify the imbalance of carbon and nitrogen (N) being delivered to rumen microbes, thereby making N utilization by the microbes more efficient and decreasing N loss from the animal (Ellis et al., 2010). The candidate gene TSub_g18549 (lectin receptor protein kinases) identified in the present study may be binding various disaccharides and other complex sugars and thereby reducing their availability to ruminal microorganisms and methanogenic potential. Being in the haplotype block, these two genes are the most stable and promising potential candidates to design molecular markers to track haplotype variation for this complex trait and warrant further functional validation in these aspects. The IVFT bioassay used in the present study is a 24 h incubation and does not take into account potential changes and adaption in rumen microflora that might modify the initial affect. To validate this further, longer term *in vitro* studies using the rumen simulation technique (RUSITEC) to better mimic what may be occuring *in vivo* are recommended for phenotyping this complex trait while functionally validating the candidates identified in the present study.

The origin of the assessed germplasm spans the Mediterranean basin from Morocco and the Iberian Peninsula in the west to Turkey and Israel in the east and an altitudinal range from 3 to 1300 m asl. Some traits, such as flowering time, are strongly associated with climatic and other environmental variables over this broad range of ecological conditions indicating their adaptive nature (Abdi, 2015). Other traits, such as methanogenic potential, unrelated to any environmental variable associated with the collection location, are clearly non-adaptive.

This research creates a new paradigm to mitigate methane production by ruminants, which is one of the most significant challenges to global livestock production. Until now, the three main strategies for mitigating methane from ruminants without compromising animal productivity and profitability have focused on manipulating the host animal through animal breeding, the feed by changing diet composition (e.g., increasing the level of concentrate vs. forage), or the rumen microbial population responsible for generating the methane through the use of feed additives (Hegarty et al., 2010). Our work demonstrates that it is possible to use a targeted plant breeding approach to enhance the global effort to mitigate enteric methane emissions, without compromising animal productivity. Coupled with the development of extensive genomic resources, the intermediate heritability for the trait makes it an ideal candidate for MAS and provides an environmentally friendly model approach to mitigate methane for the genus *Trifolium* and other legumes. The genomic and molecular data from the present study can now be exploited for analysis of genetic diversity and trait-dissection, as well as gene tagging for MAS. Phenotyping for methanogenic potential is costly and uses fistulated sheep. The availability of diagnostic markers based on the suite of genes identified will minimize the need for animals in the screening process. A similar approach in the development of diagnostic markers for MAS in subterranean clover and related species can be applied to other economic traits, such as phosphate-use efficiency and oestrogenic isoflavone content (Nichols et al., 2013).

## Conclusion

Our results contribute significantly to meeting the global challenge for ruminant livestock industries to improve productivity and production efficiency while also reducing their environmental footprint. The suite of genes identified through GWAS (7) and QTL analyses (11) form the basis of a resource for use in breeding to modify plant feed composition and influence the amount of methane generated from fermentation by ruminal microbes, while also improving feed efficiency. The analyses demonstrate the feasibility of selection for haplotypes of alleles within identified genes to move the genetic constitution of pastures toward lower enteric methane production.

## Author Contributions

PK performed the research under the guidance of RA and WE and wrote the manuscript with contributions from PB, SI, HH, GK-G, JW, KaS, PV, KeS, ZD, PN, DE and CR, PK, WE, PV, ZD, PN and KeS designed the IVFT experiments, and PK performed the experiments. KeS provided the statistical expertise and conducted the analyses for the IVFT methanogenic potential data. PK, RA, SI and WE designed the sequencing experiments, and PK, SI and KaS performed the experiments. PB HH and GK-G did the bioinformatics analysis. JW provided the support and expertise on the QTL IciMapping software used to develop the next-generation linkage map and QTL analyses for the study. All authors read the manuscript and approved the content.

## Conflict of Interest Statement

The authors declare that the research was conducted in the absence of any commercial or financial relationships that could be construed as a potential conflict of interest.

## Footnotes

1 http://www.R-project.org/

2 http://www.multiqtl.com/

3 http://www.isbreeding.net

## Supplementary Material

FIGURE S1 PCA plots showing the population structure for GWAS of two sub-populations: the first sub-population comprised 27 cultivars released in Southern Australia for grazing; while the second sub-population of 97 accessions was a core germplasm collection – a stratified sample of the world collection of *Trifolium subterraneum*.

FIGURE S2 **(A)** Analysis of variance one-way (ANOVA) test among methanogenic potential (mL/g DM) measurements and non-continuous variable core vs. cultivars. **(B)** Analysis of variance one-way (ANOVA) test among methanogenic potential (mL/g DM) measurements and non-continuous variable subspecies. **(C)** Analysis of variance one-way (ANOVA) test among methanogenic potential (mL/g DM) measurements and non-continuous variable country of origin.

FIGURE S3 Correlations between methanogenic potential (mL/g DM) measurements with continuous variables (latitude, longitude, altitude, soil pH, and BioClim variables).
