# Supplementary figures and images for: Climate Clever Clovers: New Paradigm to Reduce the Environmental Footprint of Ruminants by Breeding Low Methanogenic Forages Utilizing Haplotype Variation
Source: Front Plant Sci. 2017 Sep 5;8:1463. doi: 10.3389/fpls.2017.01463 (PMC5591941; doi:10.3389/fpls.2017.01463)

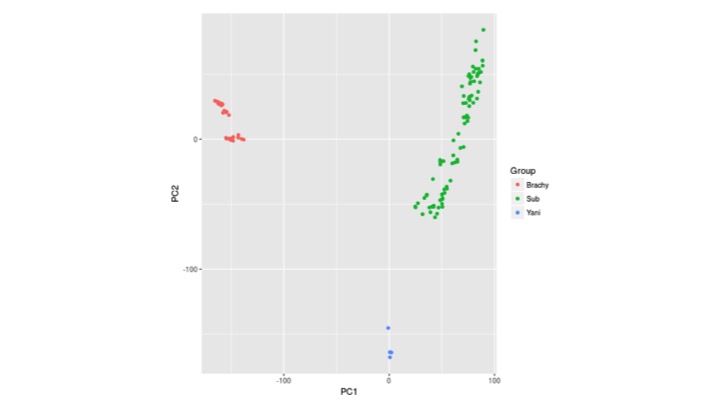

Supplement: FIGURE S1 — PCA plots showing the population structure for GWAS of two sub-populations: the first sub-population comprised 27 cultivars released in Southern Australia for grazing; while the second sub-population of 97 accessions was a core germplasm collection – a stratified sample of the world collection of Trifolium subterraneum. [file Image_1.JPEG]

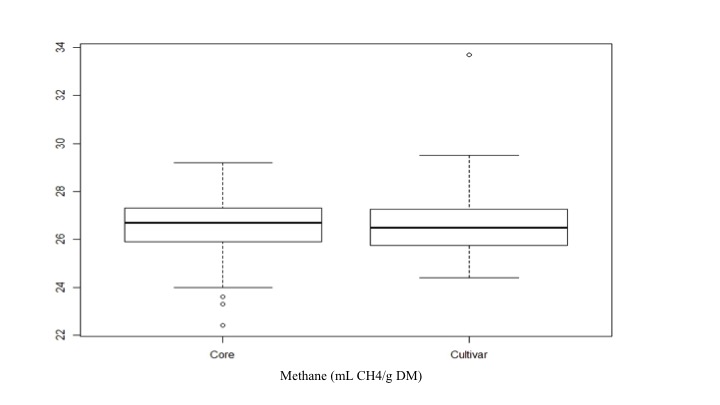

Supplement: FIGURE S2 — (A) Analysis of variance one-way (ANOVA) test among methanogenic potential (mL/g DM) measurements and non-continuous variable core vs. cultivars. (B) Analysis of variance one-way (ANOVA) test among methanogenic potential (mL/g DM) measurements and non-continuous variable subspecies. (C) Analysis of variance one-way (ANOVA) test among methanogenic potential (mL/g DM) measurements and non-continuous variable country of origin. [file Image_2.JPEG]

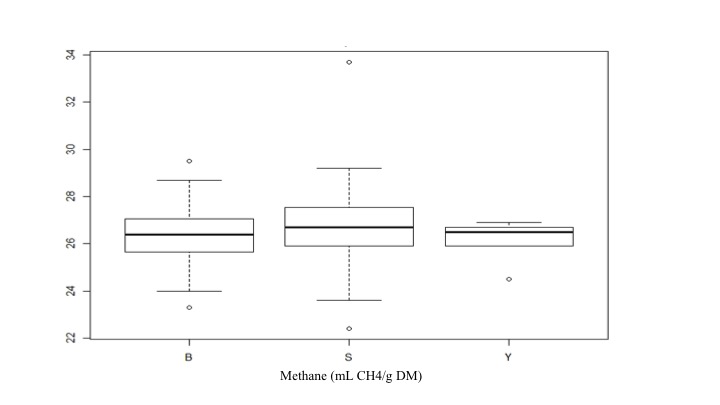

Supplement: FIGURE S3 — Correlations between methanogenic potential (mL/g DM) measurements with continuous variables (latitude, longitude, altitude, soil pH, and BioClim variables). [file Image_3.JPEG]

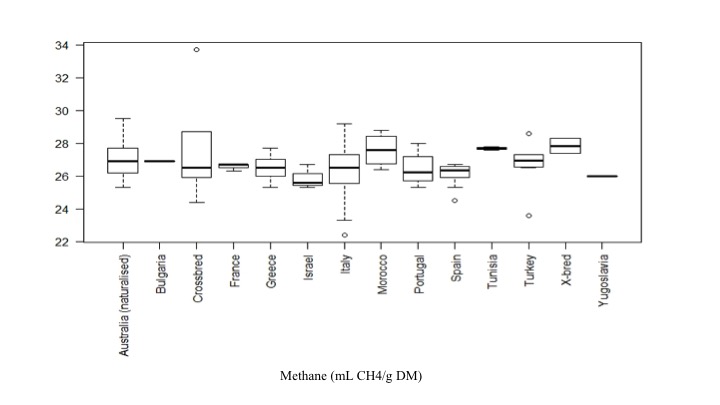

Supplement: Supplementary file 4 [file Image_4.JPEG]

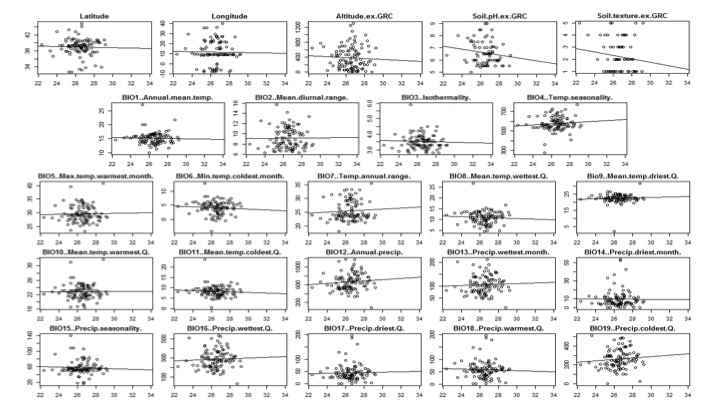

Supplement: Supplementary file 5 [file Image_5.JPEG]
